# Morphological and Thermographic Factors of the Lower Limbs Before Competition and Their Impact on Performance at the Spanish National Cross Country Championships

**DOI:** 10.3390/bios16070369

**Published:** 2026-07-07

**Authors:** Alessio Cabizosu, Victor Ruiz-Angui, Carmen Carazo-Díaz, Francisco Javier Martínez-Noguera, Pedro E. Alcaraz

**Affiliations:** 1 THERMHESC Group, San Antonio Catholic University of Murcia, 30107 Guadalupe, Spain; vruiz08@alu.ucam.edu; 2 Department of Biostatistics, Catholic University of Murcia (UCAM), 30107 Murcia, Spain; ccarazo@ucam.edu; 3 Research Center for High Performance Sport, Catholic University of Murcia (UCAM), Campus de los Jeronimos, 30107 Murcia, Spain; fjmartinez3@ucam.edu (F.J.M.-N.); palcaraz@ucam.edu (P.E.A.)

**Keywords:** thermography, endurance performance, athletic, running

## Abstract

Introduction: Cross-country running performance is influenced by a complex interaction of physiological, biomechanical, and morphological factors. Recently, infrared thermography (IRT) has emerged as a non-invasive method to assess skin temperature (TSK) and detect potential asymmetries associated with neuromuscular status, fatigue, and injury risk. However, limited evidence exists regarding its relationship with competitive performance in endurance athletes. Methods: An observational study, conducted with STROBE guidelines, included 24 national-level cross-country athletes competing in the 2026 Spanish National Championships. Pre-competition assessments comprised bilateral thermographic analysis of the anterior and posterior thigh and leg regions, alongside some anthropometric measurements (thigh and leg circumferences) following ISAK standards. Performance was evaluated using official race times. Independent *t*-tests and linear regression models were applied to assess sex differences and associations between variables. Results: No significant sex differences were observed in thigh circumference, whereas males presented significantly greater leg volume (right *p* = 0.020; left *p* = 0.042). Thermographic analysis showed no differences in bilateral thermal asymmetry (ΔTSK) between sex quadriceps (*p* = 0.077), hamstrings (*p* = 0.695), shins (*p* = 0.510), and calves (*p* = 0.194); however, higher absolute temperatures were observed in males in specific thigh regions (right anterior *p* = 0.039, right posterior *p* = 0.015, left posterior *p* = 0.020). Males achieved significantly faster race times during the first four laps, t1 (*p* ≤ 0.001), t2 (*p* = 0.002), t3 (*p* = 0.002), and t4 (*p* = 0.008), but there was no difference in the fifth lap, t5 (*p* = 0.179). Statistically significant correlations were observed between temperature differences in the various anatomical regions and competition results during the first four laps, in three of the four regions analyzed (anterior thigh *p* = 0.035, posterior thigh *p* = 0.010, anterior leg *p* ≤ 0.001). Conclusions: Pre-competition thermal asymmetry of the lower limbs appears to be negatively associated with endurance performance, potentially reflecting suboptimal neuromuscular status or incomplete recovery. IRT represents a practical and sensitive tool for monitoring athletes’ physiological readiness.

## 1. Introduction

Cross-country is a track and field discipline consisting of endurance races held on outdoor courses on surfaces such as dirt, grass, mud, or gravel, and featuring elevation changes, natural obstacles, and variations in terrain [1]. These features impose highly variable mechanical and metabolic demands compared to track running, requiring continuous adjustments in stride mechanics, muscle recruitment patterns, and energy expenditure [2,3]. Physical performance in this sport depends on the interaction of multiple factors, including physiological, biomechanical, and morphological factors, particularly in the lower limbs, which have been the subject of numerous studies on the risk of overuse injuries [4,5]. Moreover, the irregularity of the terrain may exacerbate inter-limb asymmetries and increase neuromuscular load, potentially affecting performance and injury risk [6].

In these types of events, due to varying courses, changes in pace, and adverse weather conditions, the anatomical and physiological characteristics of athletes become particularly important for optimizing competitive performance [7]. Among the most studied factors are maximum oxygen consumption (VO_2MAX_) [1], running economy [8], body composition, and certain anthropometric variables [9] that can influence mechanical and metabolic efficiency during prolonged exercise. Additionally, the fractional utilization of VO_2MAX_ and lactate threshold have been identified as key determinants of endurance performance, particularly in competitions with stochastic pacing profiles [10,11].

The anthropometric characteristics of the lower limbs have been considered a possible factor in performance in endurance running disciplines [12,13]; in fact, according to some authors [14,15,16], depending on the competition, the length of body segments, muscle circumferences, and skinfold thicknesses can influence both running mechanics and the energy efficiency of movement. A lower percentage of body fat and an appropriate ratio of muscle mass to total body mass have traditionally been associated with better performance in long-distance and middle-distance runners [17], as they allow for the optimization of the ratio between the force generated and mass moved during locomotion. Likewise, certain structural characteristics of the lower limbs can promote more efficient biomechanical patterns, reducing the energy cost of running [18,19]. Furthermore, muscle-tendon unit properties, particularly stiffness, play crucial roles in the storage and reutilization of elastic energy, especially in uneven terrains typical of cross-country events [20].

In recent years, with the aim of providing new insights that could help optimize athletic performance, advances in non-invasive technologies have made it possible to incorporate new tools for analyzing physiological and functional status in various fields of sports medicine. Among these, IRT has emerged as a technique of growing interest in the fields of track and field, cycling, and race walking [21,22,23,24,25,26,27]. This non-invasive, reliable, objective, and relatively inexpensive tool, compared to other medical diagnostic techniques, measures skin surface temperature (TSK) by detecting infrared radiation emitted by the human body, generating thermal maps that reflect the distribution of skin temperature across different body regions [28,29]. Importantly, IRT allows for real-time assessment without interfering with athlete preparation, making it particularly suitable for pre-competition monitoring [30]. Its non-invasive nature, speed of application, and the ability to take measurements in pre-competition conditions have contributed to its use in both research and the monitoring of athletic training.

We know from other authors that TSK is closely related to various physiological processes, including blood perfusion, tissue metabolic activity, and thermoregulatory mechanisms [31,32,33]. During physical exercise, the body activates different strategies to cope with the musculoskeletal demands generated by the type of activity performed, which can alter the distribution of TSK; therefore, IT has been proposed to evaluate muscle recovery processes, injury prevention, and to analyze physiological responses to training and competition [34,35]. In the field of sports, the presence of thermal pre-competition skin temperature and subsequent performance in endurance tests, asymmetries between opposite limbs in the same individual, have been associated with neuromuscular imbalances, inflammatory processes, states of fatigue, or morphological characteristics of subcutaneous tissue [35,36,37]. Such asymmetries may reflect altered muscle activation patterns or localized circulatory changes, potentially compromising mechanical efficiency during exercise [38]. Therefore, the anthropometric characteristics of the lower limbs, particularly the thickness of subcutaneous adipose tissue, could influence heat dissipation and superficial thermal distribution [39,40,41].

Currently, despite growing interest in the application of IRT in sports, the number of studies that have examined the possible relationship between these two factors remains limited. However, some evidence suggests that thermal differences among athletes prior to competition may be related to better management of the fatigue response during the event [42]. Adipose tissue acts as an insulator, potentially attenuating heat transfer from deeper tissues to the skin surface, thereby affecting TSK measurements [43].

Understanding this relationship could provide valuable insights into pre-competition physiological status and contribute to the development of additional tools for monitoring athletic performance. Furthermore, the combined analysis of thermographic and anthropometric variables could offer a more comprehensive view of the factors influencing performance in endurance athletes. This integrative approach could help identify athlete-specific profiles related to efficiency, fatigue resistance, and thermoregulatory capacity. The interaction between lower limb morphology and TSK could reflect specific physiological adaptations related to training and metabolic efficiency. Furthermore, the assessment of these variables under pre-competition conditions could help identify patterns associated with better performance in competition.

Cross-country running provides a particularly interesting context for this type of research due to the high physiological and biomechanical demands placed on the lower limbs and the ecological validity of the competition setting. Unlike laboratory-based assessments, cross-country competitions involve complex, real-world conditions that challenge both physiological regulation and mechanical efficiency, making them ideal for studying integrated performance determinants [3].

In this context, the objective of this study was to analyze the possible relationship between skin temperature in the lower limb assessed using IRT and lower limb circumference measured before the competition phase, in relation to the performance subsequently achieved by professional athletes during the Spanish Cross Country Championships. Our hypothesis is that pre-competition resting body temperature may correlate with the times achieved during the race.

## 2. Methods

### 2.1. Study Design

An observational study was conducted, based on the Strengthening the Reporting of Observational Studies in Epidemiology (STROBE) guidelines [44], with national-level professional athletes at the 2026 Spanish Cross Country Championships. This study was carried out over three separate sessions: the first for familiarization, the second for collecting thermographic data and limb circumference measurements, and finally the third for conducting the test. A repeated-measure pre-competition assessment design was employed to evaluate the potential association between baseline physiological variables and subsequent competitive performance under ecological conditions.

Pre-competition results were correlated with times (t) recorded during the competition. Participants completed different races within the same course. Nine runners completed the 5220 m race, ten runners completed the 6981 m race, and finally, five athletes completed the 8741 m race. The thermographic patterns of the anterior thigh (quadriceps region), posterior thigh (hamstrings region), anterior leg (tibialis region), and posterior leg (triceps surae region), as well as thigh and leg circumferences, were evaluated bilaterally in relation to the time and distance achieved in the test.

This study was conducted in accordance with the Declaration of Helsinki [45] for research involving human subjects and was approved by the Ethics Committee of the Catholic University of Murcia (CE102102). All participants were informed of the study procedures and signed an informed consent form. In the case of underage athletes, their parents were informed, and written consent was obtained from their legal guardians. No financial incentives were provided. All measurements were performed between 7:30 a.m. and 9:00 a.m. to minimize the potential influence of circadian variations on skin temperature and cardiovascular variables.

### 2.2. Sample Characteristics

Twenty-four national-level athletes were recruited for this study (Table 1). The inclusion criteria for this study were as follows: being between 17 and 30 years of age; having a body mass index (BMI) between 18.0 and 25.5 kg·m^2^; having at least 2 years of experience in training and competing in track and field at the national level; and being able to understand the study guidelines and information. Athletes with any cardiovascular condition or injury and athletes who did not comply with the Thermographic Imaging in Sports and Exercise Medicine (TISEM) protocol recommendations were excluded [46]. Additionally, athletes were instructed to avoid caffeine, alcohol, intense physical exercise, massage, and the application of topical products for at least 24 h before thermographic assessment, in accordance with current IRT guidelines. The sample included both male (n = 12) and female (n = 12) athletes competing at the national level, thereby allowing the exploration of sex-related differences in thermographic and anthropometric variables.

### 2.3. Procedure

**Figure 1:**
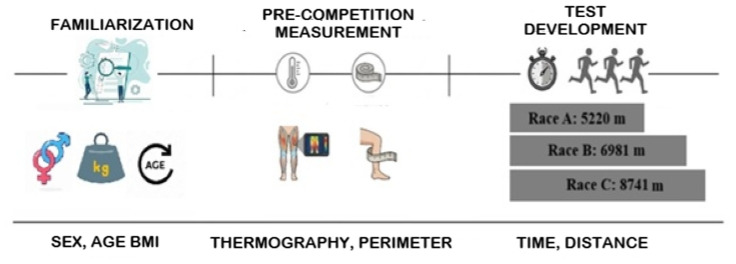
Sequence of protocol: Visits 1 and 2 and competition.

During the pre-competition orientation session, the athletes underwent a routine examination (peripheral oxygen saturation, blood pressure measurement, age, height, BMI, etc.). In addition, they received instructions on the TISEM protocol [46] and familiarized themselves with the thermographic and circumference measurement protocol that would be carried out immediately before the various tests the following day. During the second visit, TSK tests were performed on the lower limbs, and circumferential measurements of the thigh and leg were taken bilaterally. Finally, the test was conducted (Figure 1). All pre-competition measurements were completed before the warm-up routine to avoid exercise-induced alterations in skin temperature distribution.

### 2.4. Tests Performed

#### 2.4.1. Instrumentation

A Hikmicro M60 camera (Hangzhou Hikmicro Sensing Technology Co., Ltd., Hangzhou, China) was used for the thermographic study. This thermal imager has a thermal sensitivity of less than 0.04 °C and a temperature range of −20 °C to +150 °C. The emissivity was set to 0.98, as suggested by other studies [47,48]. The athletes stood in their underwear, shirtless and barefoot, on a 1.5 cm cotton pad to prevent temperature changes.

The principal investigator turned on the equipment one hour before the first recording, which took place at 7:35 a.m., and placed it on a tripod one meter away from the measurement area, at a 10° angle. The same investigator performed all image acquisitions to reduce inter-observer variability. All participants underwent a standardized acclimatization period of 15–20 min in a 20 m^2^ room with an average temperature of 22 °C (between 21 and 23 °C), humidity of 40% ± 5%, and atmospheric pressure of 1 atm. In this phase, following the acclimatization period, the first thermographic data corresponding to the pre-competition baseline were recorded.

**Figure 2:**
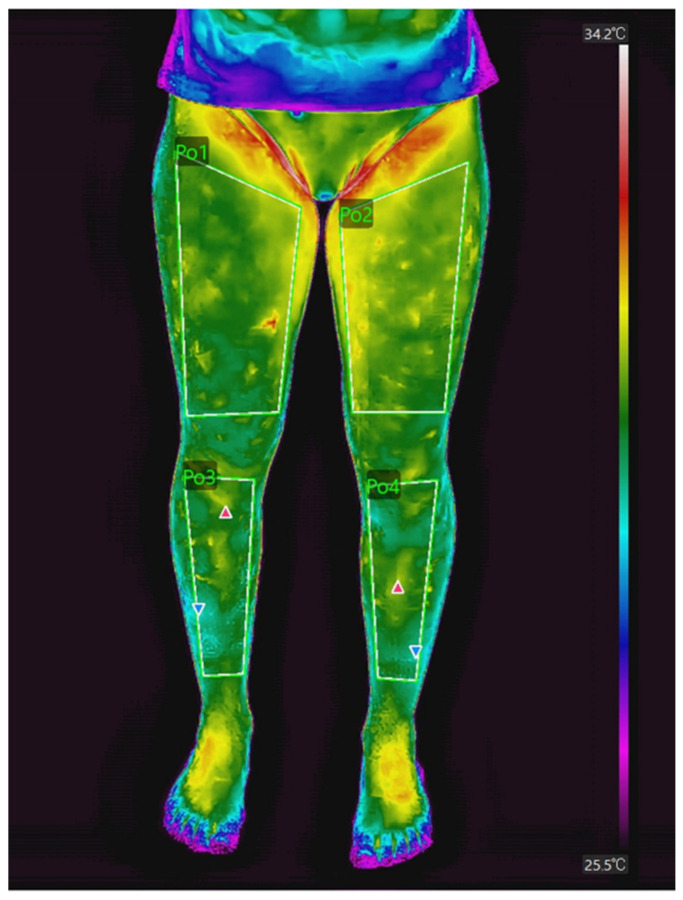
Thermography image of anterior lower extremity. Po1: right thigh (quadriceps region); Po2: left thigh (hamstrings region); Po3: right leg (tibialis region); Po4: left leg (triceps surae region).

To improve the accuracy and reliability of the analysis of anatomical regions, in all measurements, the regions of interest (ROIs) in the lower extremities were defined using standardized anatomical references described in the literature, aiming to include as many pixels as possible in each ROI [22]. Thermographic measurements were performed from both anterior and posterior views following the same model and specifications used in previous similar studies. The following areas were segmented: Anterior thigh (quadriceps region), Posterior thigh (hamstrings region), Anterior leg (tibialis region), and Posterior leg (triceps surae region) (Figure 2). Mean skin temperature (TSKmean) and bilateral thermal asymmetries (ΔTSK) were subsequently calculated for each ROI.

This approach allowed for adherence to the anatomical boundaries of the structure in the thermographic images, improving the accuracy of the image analysis. Image processing was performed by two blinded researchers using the Hickmicro Analyzer (Hangzhou, China) software (V2.1.2_260408). Inter-rater agreement was verified before statistical analysis to ensure measurement reliability. Inter-rater agreement was determined by analyzing the mean temperature values obtained in relation to the cm^2^ of the measured areas. If the temperature difference exceeded 0.5 within the same anatomical region and for the same measured area, a third researcher evaluated the images, and a consensus was reached among the three. The involvement of a third reviewer was never necessary.

For the study measuring lower limb circumference, the RENPHO smart measuring tape (Joicom Corporation, Eastvale, CA, USA) was used, a digital device designed to measure and record body circumferences and track changes in body composition over time via a mobile app. Measurement range: approximately 1 mm–1500 mm (150 cm). Accuracy: approximately ±3 mm. Approximate weight: 100 g. To ensure the reproducibility and standardization of circumference measurements, the protocol of the International Society for the Advancement of Kinanthropometry (ISAK) was used [49].

The mid-thigh crease was used as a reference point: located halfway between the anterior superior iliac spine and the top of the patella. Thigh circumference: measured around the mid-thigh crease, with the subject standing and the muscle relaxed. Point of maximum calf circumference: the widest region between the knee and the ankle is measured. The subject must be standing, with weight distributed evenly and the muscle relaxed, to ensure consistency.

#### 2.4.2. Characteristics of Court and Environmental Conditions

The 2026 Spanish Cross Country Championship race was held in Almodóvar del Río (Córdoba) on 25 January 2026, on the “El Pinillo” course. This course combines flat sections, sharp turns, and gentle slopes. As for weather conditions, the competition took place in the middle of winter, with cold temperatures ranging from 7.3 °C to 12.3 °C, a humidity level of 46%, and winds of up to 26 km/h, as reported by the State Meteorological Agency (AEMT) [50]. These environmental conditions were considered relevant due to their potential influence on thermoregulatory responses and skin temperature distribution during endurance exercise.

The races consisted of a course within a closed circuit comprising two laps of different lengths adapted for weather reasons: one 1100 m section and another 1800 m section, which were combined to complete the different distances of the races scheduled for each category. These races were approved by the Real Federación Española de Atletismo (RFEA) [51], included in the national or regional calendar, and held in the presence of judges.

#### 2.4.3. Statistical Analysis

Data analysis was performed using IBM SPSS Statistics software (v. 26.0; IBM Corp., Armonk, NY, USA) and R software (v. 4.6.0; R Core Team, Vienna, Austria). Data are presented as means ± SD. Homogeneity and normality of the data were tested using the Levene and Shapiro–Wilk tests, respectively.

Comparisons between sexes were explored using *t*-tests for independent samples. Associations between the dependent variable: Time of performance (t), and the individual predictors: temperature (Tº) or difference in temperature (ΔTº) and circumferences (CM) were explored using simple linear regression models, optimizing the fit using the Gaussian least squares method and assuming a normal distribution for the values of the dependent variable conditioned on each value of the explanatory variable.

Subsequently, multiple linear regression analyses were performed to estimate adjusted effects, taking into account potential confounding factors. Model assumptions were evaluated, including linearity, homoscedasticity, and normality of the residuals. All statistical tests were two-sided, and rather than relying on arbitrary significance thresholds (e.g., *p* < 0.05 or *p* < 0.01), *p*-values were reported as exact values to four decimal places to improve transparency and interpretability. This approach is consistent with current recommendations that discourage dichotomous interpretations of statistical significance [52,53].

## 3. Results

### 3.1. Circumferences

**Figure 3:**
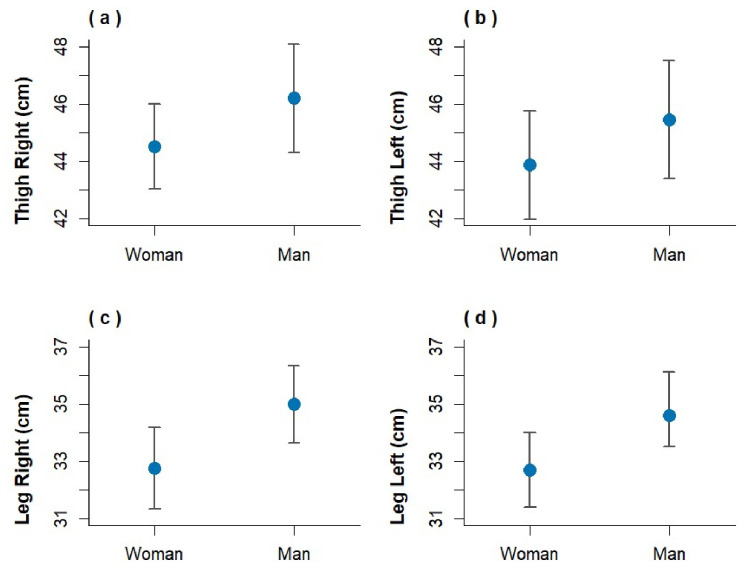
Differences in anatomical perimeters between men and women. Mean and CI 95%. (**a**) Right Thigh, (**b**) Left Thigh, (**c**) Right Leg, (**d**) Left Leg.

The independent-samples *t*-test analysis (Table 2) showed no differences in thigh volume between men and women (right *p* = 0.140; left *p* = 0.225) but did show differences in leg volume (right *p* = 0.020; left *p* = 0.042), with men having greater volume than women. These findings suggest that sex-related differences in anatomical perimeters in this cohort were more pronounced at the calf level than at the thigh level, potentially reflecting differences in lower-leg musculature and body composition associated with endurance performance. Figure 3 illustrates the circumference differences between sexes.

### 3.2. Temperature

**Figure 4:**
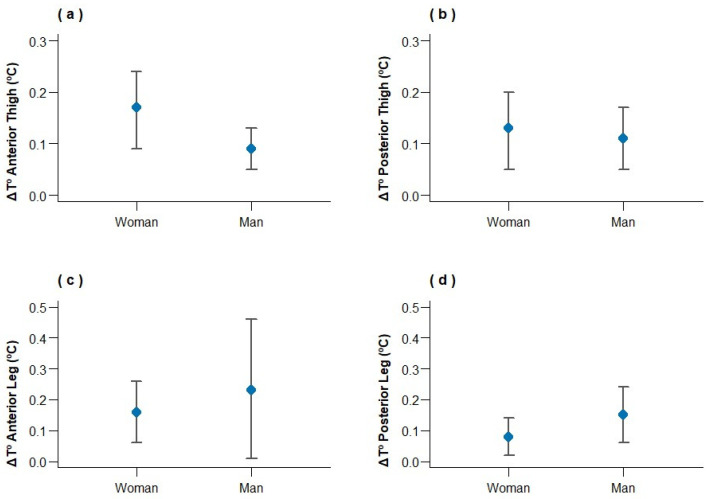
Differences between the means of the thermal differences in both limbs based on sex. Mean and CI 95%. (**a**) Anterior Thigh, (**b**) Posterior Thigh, (**c**) Anterior Leg, (**d**) Posterior Leg.

No significant differences were observed between the means of the temperature differences (ΔT) between the two limbs according to sex: quadriceps (*p* = 0.077), hamstrings (*p* = 0.695), shins (*p* = 0.510), and calves (*p* = 0.194); see Figure 4. However, in both thighs, by sex, statistically significant differences were observed in both the mean temperatures (right anterior *p* = 0.039, right posterior *p* = 0.015, left posterior *p* = 0.020) and the regional maximum temperature point (left anterior *p* = 0.035), with temperatures consistently higher in men. No significant differences were observed between the mean leg temperatures, based on sex, neither posteriorly (right *p* = 0.240, left *p* = 0.208) nor anteriorly (right *p* = 0.980, left *p* = 0.565). Overall, although thermal asymmetry patterns were comparable between sexes, male athletes tended to exhibit higher absolute thigh skin temperatures under pre-competition resting conditions. These differences may reflect sex-related variations in muscle mass distribution, blood perfusion, or thermoregulatory responses.

### 3.3. Competition Results

Based on sex, differences in race times were observed in the first four laps, t1 (*p* ≤ 0.001), T2 (*p* = 0.002), t3 (*p* = 0.002), and t4 (*p* = 0.008), but there was no difference in the fifth lap, t5 (*p* = 0.179). Men had better times for the same distance covered in four of the five laps (Table 3). The magnitude of these differences was greater during the initial stages of the race and progressively decreased in the later laps, suggesting a potential convergence in pacing strategy and fatigue development between sexes as the competition progressed.

Statistically significant correlations were observed between temperature differences in the various anatomical regions and competition results during the first four laps, in three of the four regions analyzed (anterior thigh *p* = 0.035, posterior thigh *p* = 0.010, anterior leg *p* ≤ 0.001); however, no correlations were observed in the posterior leg *p* = 0.247, with generally worse lap times observed as thermal asymmetry between sides increased.

In general, greater bilateral thermal asymmetry was associated with slower lap times. This relationship was particularly evident in the anterior leg region, where athletes presenting larger ΔTSK values tended to demonstrate poorer performance during the early and intermediate phases of the race.

**Figure 5:**
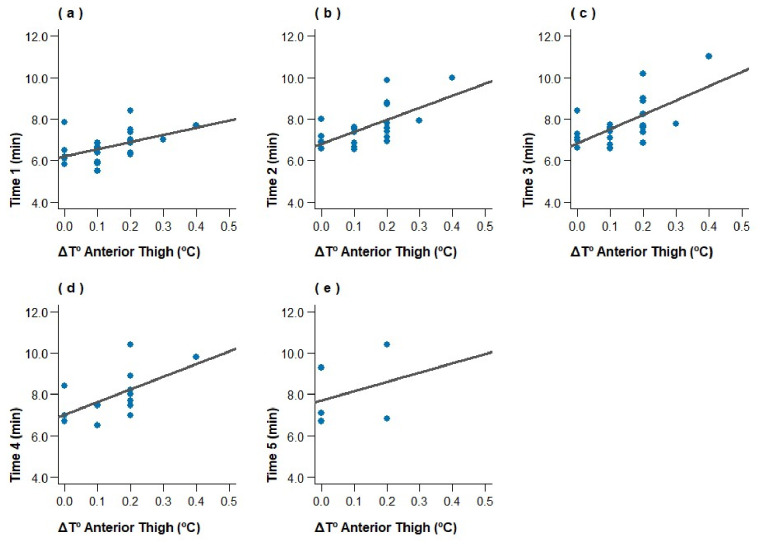
Correlation between times and temperature differences in the anterior thigh region. (**a**) Time 1, (**b**) Time 2, (**c**) Tome 3, (**d**) Time 4, (**e**) Time 5.

Figure 5 shows the relationship between pre-competition anterior thigh thermal asymmetry and race performance. Regardless of sex, athletes exhibiting greater bilateral thermal differences before competition obtained slower lap times across t1–t5. These findings suggest that pre-competition thermal asymmetry may reflect altered physiological or neuromuscular status capable of negatively influencing endurance performance.

The graphs show that, regardless of gender, athletes who exhibited greater asymmetry between both sides prior to the competition (ΔT anterior thigh) achieved worse times during the test (t1, t2, t3, t4, t5). Moreover, the absence of significant associations in the posterior leg region may indicate a lower sensitivity of this anatomical area to pre-competition physiological disturbances or a reduced contribution to pacing variability during competition.

## 4. Discussion

The objective of the present study was to examine the association between some anthropometric aspects, thermographic variables of the lower limbs and physical performance before competition, during the Spanish National Cross Country Championships in national-level athletes. Overall, the main findings indicate that: (I) sex-related in anatomical perimeters differences were observed primarily at the calf level rather than at the thigh level; (II) men exhibited higher absolute skin temperatures in several thigh regions, although no sex differences were found in bilateral thermal asymmetry (ΔTSK); and (III) greater pre-competition thermal asymmetry was associated with poorer performance during the initial and intermediate stages of the race. Taken together, these findings support the hypothesis that pre-competition thermal status may reflect underlying physiological and neuromuscular conditions relevant to endurance performance.

### 4.1. Circumferences and Temperature

Regarding anatomical perimeter variables, our studies confirmed findings previously reported by other authors [54,55], which showed that at the thigh level, for the same BMI, no differences in circumference were observed between men and women, but differences were observed at the lower leg level. This observation may reflect regional differences in muscle distribution and adipose tissue accumulation between sexes. Men generally present greater muscle mass and a lower percentage of subcutaneous adipose tissue compared to women, with a general consensus that fat distribution in women tends to accumulate in a higher percentage in the proximal region of the lower limb (gluteal-femoral region) compared to more distal levels [56]. Therefore, in our study, although there may be muscular differences in the lower extremities between the sexes, these differences could be compensated for by the amount of subcutaneous adipose tissue at the proximal level, whereas at the distal level, where the concentration of adipose tissue tends to be equal, muscle size is the predominant factor, being greater in men than in women. This finding would also explain the thermographic results obtained regarding absolute temperatures based on sex. Our results are consistent with previous studies conducted in healthy individuals, where other authors found higher skin temperatures in the leg muscles in males [57]. On the one hand, these differences have been attributed primarily to variations in resting metabolic activity [58,59,60,61], muscle blood flow [61,62], and the production of certain hormones [63]; however, the higher percentage of subcutaneous adipose tissue in women may act as a thermal insulator, reducing heat transfer from the muscle to the skin surface, which could influence basal thermal regulation [64,65]. Furthermore, it is worth noting that, in our study, no thermal differences between sexes were observed in the anterior and posterior regions of the leg, where there are typically no differences in fat concentration between genders; however, volumetric differences were observed, since in this region the difference in muscle size would not be compensated for by subcutaneous fat. Our results therefore suggest that the thermographic response at the distal level may be more closely related to patterns of functional activation than to morphological or sex-related differences. From an applied perspective, these results may be relevant for coaches and sports medicine practitioners seeking to monitor muscular readiness and recovery using IRT under ecological pre-competition conditions.

### 4.2. Temperature and Performance

Regarding the ΔTSK between sides at rest, our results support the findings of previous studies, in which, regardless of gender, no thermal asymmetries exceeding 0.5 °C were observed in any of the anatomical regions analyzed. This is because, in both healthy individuals and trained, injury-free athletes, the thermal distribution between both sides of the body tends to be highly symmetrical [57,66]. It is known from other authors that, in healthy individuals, thermal asymmetries greater than 0.5 °C are often associated with muscle overload or injury [67], while there is evidence that in athletes this threshold can drop to 0.3 °C, with a homogeneous thermal distribution reflecting adequate functional and neuromuscular balance [68].

In this regard, our results demonstrate a correlation between greater pre-competition thermal asymmetries and poorer competition times in the first four laps, providing relevant information on the role of pre-exercise physiological state in athletic performance. These results align closely with the main objective of the study and reinforce the potential value of IRT as a non-invasive tool for assessing pre-competition physiological readiness. From a physiological perspective, the presence of pre-competition thermal asymmetries could reflect inadequate recovery or poor assimilation of the training load [69], which negatively affects competition performance; therefore, it is plausible that athletes with greater asymmetries begin the competition in a suboptimal state [70]. Even in the absence of overt injury, such disturbances may compromise movement efficiency, increase metabolic cost, and negatively affect pacing capacity during competition [71]. The fact that a correlation could only be established for the first four laps may be because only four athletes completed lap five, meaning that such a small sample size could influence the statistical results obtained.

The stronger associations observed during the initial laps may indicate that pre-competition physiological status exerts a greater influence during the early phases of endurance performance, when pacing strategy and neuromuscular freshness are particularly relevant. As the race progresses, additional factors such as accumulated fatigue, tactical decisions, and substrate depletion may progressively reduce the influence of baseline thermal status on performance outcomes.

No significant relationship was observed in the posterior leg region. This absence of association could indicate lower sensitivity of the triceps surae thermal profile to pre-competition physiological disturbances or may reflect the smaller contribution of this region to inter-individual pacing variability in the present competition context. However, the reduced number of athletes completing the fifth lap may also have limited the statistical power of the analysis.

Future studies could examine the relationship between pre-competition thermal asymmetry and markers of muscle fatigue during and after competition to further clarify this aspect. This could be of great help to physical trainers seeking to optimize their athletes’ performance, as it expands the applicability of IRT in load monitoring and decision-making within a competitive context, especially in sports where the margin for improvement is narrow.

### 4.3. Performance and Sex Difference

About performance throughout the race, men achieved significantly better lap times during the first four laps, while by the fifth lap, the differences were no longer statistically significant. This pattern is consistent with the literature describing higher values of muscle power [72,73], maximum oxygen consumption [74], and anaerobic capacity [75,76] in men, factors that typically translate into higher running speeds during the initial phases of high-intensity exercise.

The progressive reduction in sex-related performance differences throughout the race may also reflect differences in pacing strategy and fatigue resistance. However, the narrowing of the gap in the final lap could be related to the onset of fatigue or to differences in pacing strategies between the sexes, as previous research has indicated that women tend to adopt more conservative pacing strategies in the early stages of the competition [77,78], which may lead to more consistent performance in the final stages, compared to men.

### 4.4. Limitations and Strengths

Despite the promising results, we believe these data should be interpreted with caution due to the limitations of our study. First, IRT is a technique that is highly sensitive to external variables (ambient temperature, hydration, stress, etc.), which makes it difficult to obtain and extrapolate comprehensive and fully replicable results, especially in a sample of athletes as small as ours. Second, the observed relationship is correlational; therefore, a direct causal relationship cannot be established between pre-competition thermal asymmetries and performance during the race. Third, it is possible that both variables are mediated by a third factor, such as fatigue or accumulated training load; therefore, it would be interesting in future studies to take post-competition measurements of all variables. A fourth consideration might be the standardization of ROIs. Since this is performed manually, it is difficult to avoid including unwanted anatomical structures in the analysis, which could potentially skew the average thermal results obtained. Future studies should include longitudinal designs and combine IRT with other variables (internal load, perceived fatigue, biomarkers) and with other techniques (electromyography, ultrasound, or spectroscopy) to better understand the underlying mechanisms. In addition, it would be advisable to increase the sample size, including a wider range of ages and BMI, especially for the final stages of the study, to ensure greater statistical robustness and allow for more accurate comparisons between the two sexes across all variables. Likewise, pacing strategies could be analyzed in greater detail, incorporating variables such as segment-specific pace, subjective perception of effort, and the participants’ physiological profiles, with the aim of better understanding differences in effort distribution throughout the competition. We also believe it would be of interest to include direct physiological measurements, such as oxygen consumption, lactate concentration, or heart rate, in conjunction with thermographic examinations, to more accurately relate observed performance to the underlying mechanisms of fatigue and the associated thermographic response. In conclusion, the evidence suggests that pre-competition thermal asymmetries could serve as a useful marker of an athlete’s functional status and may be associated with poorer performance in competition. This reinforces the value of IRT as a comprehensive monitoring tool, not only for injury prevention but also for optimizing athletic performance.

## 5. Conclusions

The results observed in this study demonstrate that the IRT value, before competition, could be a valid tool for measuring, analyzing, and quantifying pre-competition tissue responses, linking changes in skin temperature to the responses observed during competition. From an applied perspective, the evaluation of muscle thermal patterns can provide relevant information on muscle activation, load distribution, and potential injury risk, which has led to its growing use in the fields of sports medicine and training monitoring. Accordingly, IRT could complement existing athlete monitoring strategies in sports medicine and performance science. Future studies should investigate the interaction between thermographic responses and additional physiological, metabolic, and neuromuscular variables in order to better understand the mechanisms underlying the relationship between skin temperature and endurance performance.

## Figures and Tables

**Table 1:** Baseline characteristics of the sample.

				CI 95%				
	Sex	N	Mean	Lower	Upper	SD	Min	Max
Age (years)	W	12	20.1	18.0	22.1	3.2	17	29
	M	12	20.1	18.0	22.2	3.3	17	27
Weight (Kg)	W	12	52.5	49.3	55.7	4.9	44.3	59.1
	M	12	62.3	58.5	66.2	5.9	54.0	76.1
Hight (Cm)	W	12	161.0	157.2	164.8	6.0	153	173
	M	12	175.3	169.8	180.9	8.7	162	195
BMI	W	12	20.3	19.4	21.2	1.4	18	23
	M	12	20.3	19.2	21.3	1.7	18	23
Systolic (mmHg)	W	12	107.0	99.5	114.5	11.8	84	119
	M	12	116.2	114.9	117.4	1.9	113	118
Diastolyic (mmHg)	W	12	64.8	59.7	69.8	7.9	49	75
	M	12	67.9	65.8	70.0	3.3	62	74
Heart rate (bpm)	W	12	61.5	57.4	65.6	6.5	48	69
	M	12	57.8	54.9	60.8	4.6	51	67
pO_2_ (%)	W	12	98.8	98.5	99.0	0.4	98	99
	M	12	98.8	98.6	99.1	0.3	98	99

BMI: body mass index, CI 95%: 95% confidence interval, M: Man, W: Woman, pO_2_: partial pressure of oxygen, SD: standard deviation.

**Table 2:** Differences in anatomical perimeters between men and women.

CI 95%					
	MD	SE	Lower	Upper	*p*-Value
Thigh Right	1.67	1.09	−0.59	3.94	0.140
Thigh Left	1.59	1.27	−1.05	4.22	0.225
Leg Right	2.24	0.89	0.38	4.09	0.020 *
Leg Left	1.84	0.85	0.08	3.61	0.042 *

CI 95%: 95% confidence interval, MD: Mean Difference, SE: Standard Error of the Mean. * *p* < 0.05.

**Table 3:** Time reference data by gender.

				CI 95%					
Time	Sex	N	Mean	Lower	Upper	SD	Min	Max	*p*-Value
T1 (min)	W	12	7.2	6.8	7.6	0.6	6.2	8.4	<0.001 ***
	M	12	6.2	5.9	6.4	0.3	5.5	6.7	
T2 (min)	W	12	8.1	7.5	8.8	1.0	6.9	10.1	0.002 **
	M	11	7.0	6.7	7.3	0.4	6.5	7.6	
T3 (min)	W	12	8.4	7.6	9.2	1.2	7.0	11.0	0.002 **
	M	11	7.0	6.8	7.3	0.4	6.6	7.6	
T4 (min)	W	8	8.6	7.6	9.5	1.1	7.0	10.4	0.008 **
	M	7	7.2	6.8	7.6	0.4	6.5	7.5	
T5 (min)	W	3	8.9	4.8	13.1	1.7	7.1	10.4	0.179
	M	2	6.8	6.0	7.5	0.1	6.7	6.82	

CI 95%: 95% confidence interval, M: Man, W: Woman, SD: Standard Deviation. ** *p* < 0.01, *** *p* < 0.001.

## Data Availability

The original contributions presented in this study are included in the article. Further inquiries can be directed to the corresponding author.
